# Exploring Factors Impacting on the Lane Choice of Riders of Non-Motorized Vehicles at Exit Legs of Signalized At-Grade Intersections

**DOI:** 10.3390/ijerph18126327

**Published:** 2021-06-11

**Authors:** Guoqiang Zhang, Qiqi Zhou, Jun Chen

**Affiliations:** 1 School of Transportation, Southeast University, Nanjing 210096, China; 220193149@seu.edu.cn (Q.Z.); chenjun@seu.edu.cn (J.C.); 2 National Demonstration Center for Experimental Road and Traffic Engineering Education (Southeast University), Nanjing 211189, China

**Keywords:** behaviors of riders, non-motorized vehicles, influencing factors, signalized at-grade intersections, exit legs, binary logistic regression, probability prediction model

## Abstract

For most signalized at-grade intersections, exclusive lanes for non-motorized vehicles have been applied to improve the level of service, capacity and safety of both motorized vehicles and non-motorized vehicles. However, because of various factors, riders of non-motorized vehicles have been observed using lanes for motorized vehicles instead of lanes for non-motorized vehicles, which usually negatively influences the performance of signalized intersections and sometimes may cause serious problems such as traffic congestion and accidents. The objective of this paper is to explore factors influencing the lane choice of riders of non-motorized vehicles at exit legs of signalized at-grade intersections and develop a prediction model for riders’ lane choice. Data concerning the lane choice of riders of non-motorized vehicles and other impacting factors were collected at exit legs of four typical signalized at-grade intersections. Applying binary logistic regression, a probability prediction model was developed to explain how various factors influence the lane choice of riders of non-motorized vehicles. The prediction model indicates that female riders of non-motorized vehicles have a higher probability of choosing the lane for non-motorized vehicles than male riders. Compared with riders of non-motorized vehicles powered by electricity, riders of traditional man-powered bicycles are more likely to choose the lane for non-motorized vehicles. Right-turning riders of non-motorized vehicles are more likely to choose the lane for non-motorized vehicles than straight-going riders, who in turn, are more likely to choose the lane for non-motorized vehicles than left-turning riders. Decreasing the volume of non-motorized vehicles, increasing the volume of motorized vehicles, and widening the lane for non-motorized vehicles will increase the probability of the correct choice of lane for non-motorized vehicles. The predictions of the model are in good agreement with the observed facts. The model is meaningful for guidance on the design and management of signalized at-grade intersections.

## 1. Introduction

With the continuous development of economy and society, many Chinese cities are now experiencing fast urbanization, an enormous increase in motorized vehicles and severe problems of pollution, and the traffic supply cannot meet the need of this sharp increase in traffic demand [1]. With the confinement of limited resources of time and space, traffic congestion of urban areas has become more and more serious. Under such a background, non-motorized vehicles are now becoming very popular in many Chinese cities, as a convenient and cheap mode of transportation. Compared with motorized vehicles, non-motorized vehicles have no air pollution and can provide a more efficient and reliable service for people’s daily needs [2]. The appearance and development of electrically powered non-motorized vehicles have enlarged non-motorized vehicles’ scope of application greatly. Compared with man-powered non-motorized vehicles, they are now used for much longer distances of travel and can now meet more kinds of traffic demand. As well as being used in citizens’ daily travels for working, shopping and entertainment, electrically powered non-motorized vehicles have become one of the major means of transportation for express delivery, which is the backbone of fast-increasing e-commerce. However, the great development of non-motorized vehicles has also brought some problems, such as a more and more widespread mixture of motorized vehicles and non-motorized vehicles, which affects the safety of riders of non-motorized vehicles and the efficient movements of motorized vehicles.

**Figure 1:**
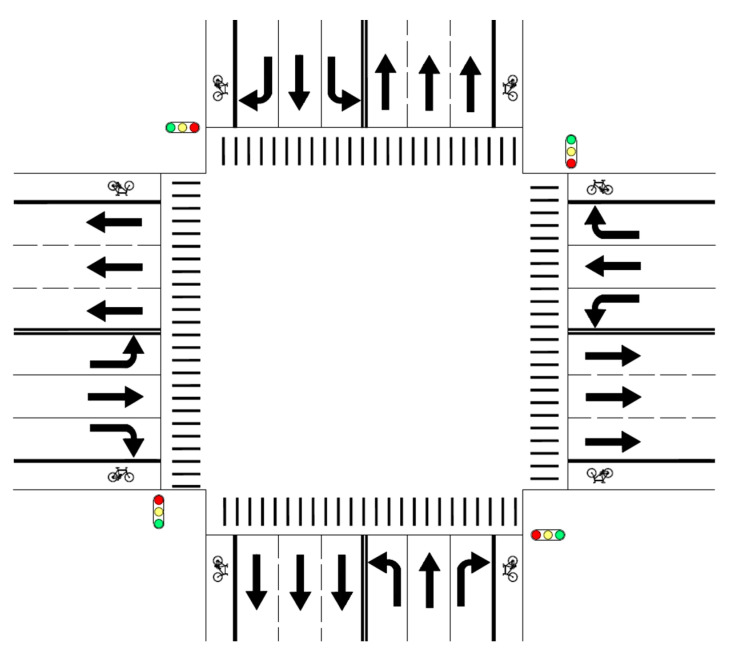
Intersection scheme with exclusive lanes for motorized vehicles and exclusive lanes for non-motorized vehicles.

As non-motorized vehicles have been widely used in China, a mixture of motorized vehicles and non-motorized vehicles is one of the major characteristics of Chinese traffic systems. It is one of the major causes of traffic accidents involving non-motorized vehicles and interferes with the movements of motorized vehicles, resulting in more serious traffic jams, higher consumption of gasoline and higher carbon dioxide and poisonous gas emissions. Therefore, it is one of the major problems currently facing Chinese urban transportation. In order to avoid or alleviate the mixture of motorized vehicles and non-motorized vehicles, designers of urban road systems have designed exclusive lanes for riders of non-motorized vehicles, which lie to the right of exclusive lanes assigned to motorized vehicles, so that motorized vehicles and non-motorized vehicles can use separate lanes (see Figure 1). However, because of the impacts of various subjective and objective factors, riders of non-motorized vehicles have been observed to encroach on exclusive lanes for motorized vehicles from time to time, though such behaviors are both illegal and risky. At exit legs of signalized at-grade intersections, where traffic conflicts are rather serious because traffic flows from different approaches have to merge when they enter exit legs at the same time, such problems are especially pronounced, which may endanger the traffic safety and efficiency of the whole road system.

It is, therefore, very urgent that behaviors of riders of non-motorized vehicles in relation to their choice of lanes at critical locations be explored, so as to establish the mechanisms behind their various risky behaviors. Under such a background, the primary objective of this paper is to investigate the behaviors of riders of non-motorized vehicles at exit legs of signalized at-grade intersections and to explore how various factors impact on their choice of lane.

## 2. Literature Review

Due to the unsteady movements of non-motorized vehicles, research has been carried out to explore the special movements of non-motorized vehicles and behaviors of their riders. Wei and Kai [3] built a series of bicycle-following models with three different BP neural network structures, considering the influences of some critical parameters in the bicycle traffic, such as the distance between bicycles, and the relative speed and acceleration of the leading bicycle. A bicycle-following model was proposed, which could better reflect the stimulus–response relationship between the leading and the following bicycles. Yan et al. [4] developed a two-dimensional mathematical model for the motion of a rider–mountain bike-coupled system, based on the multi-body system dynamics theory. The model focused on the simulation of vibration stress on the rider due to an uneven track. They designed an experimental testing method to rectify this model.

Recently, based upon a survey of riders of non-motorized vehicles, their psychology and behavior have been widely studied. Zhang et al. [5] analyzed the unsafe behaviors of cyclists, based on the theory of planned behavior. Through increasing the variables and adopting a questionnaire method, the psychological factors of cyclists were analyzed. The results indicated some problems, such as incorrect awareness of unsafe behaviors, poor law-abiding ability, and common unsafe behavior experience. Zhang et al. [6] established and tested an electric bicycle rider’s unsafe crossing behavior model, based on the theory of planned behavior (TPB). Wilbur and Schroeder [7] explored distracted bike riding and its relation to other unsafe bicycling behaviors, based on the 2012 National Survey of Bicyclist and Pedestrian Attitudes and Behavior. They found that respondents who had ridden a bicycle within the past year and who reported having used an electronic device for at least some of their rides demonstrated a higher prevalence of unsafe behaviors than those who hardly ever or never used an electronic device while riding a bicycle. Hezaveh et al. [8] reported the psychometric properties of a newly designed measurement instrument, the Bicycle Rider Behavior Questionnaire (BRBQ).

Chen et al. [9] conducted a household survey among 1244 adult non-motorized vehicle riders to compare attitudes and behaviors about non-motorized vehicle traffic safety between bicycle and electric bicycle riders. Compared to the man-powered bicycle riders, the riders of power-assisted bicycles showed a significantly lower cognition on traffic safety-related risk behaviors, especially on not wearing reflective tapes while riding at night, not wearing a helmet while riding, and installing an umbrella on power-assisted bicycles. Wang et al. [10] compiled the E-bike Rider Behavior Questionnaire (ERBQ) and obtained 573 valid questionnaires. The study indicated that male drivers had more frequent illegal behaviors and leading behaviors; with the increase of age, drivers had fewer leading behaviors, but more illegal behaviors. Lower-educated drivers had more aggressive behaviors, while high-educated drivers had more illegal behaviors. Wu et al. [11] conducted a retrospective WeChat-based online survey to examine how often shared bicycle riders reported engaging in risky cycling behaviors in urban China. Eight unsafe shared-bicycle riding behaviors were assessed. They found that shared-bicycle riders frequently engaged in some unsafe riding behaviors in urban China. Younger age, a lower level of education, and longer hours of riding each week were associated with greater risks of some unsafe riding behaviors.

Researchers have made various observations about riders of non-motorized vehicles, based upon which riders’ behaviors and impacting factors were explored. Zhang and Wu [12] studied the effect of sunshields, which are used to avoid riders suffering from sunlight and high temperature, on the red-light running behavior of cyclists and e-bike riders. Their research indicated a significant effect of sunshield on reducing the red-light infringement rate both on sunny and cloudy days. The effect of a sunshield was larger on sunny days than on cloudy days. Langford et al. [13] carried out a naturalistic GPS-based safety study between regular bicycle riders and e-bike riders in the context of a unique bike sharing system that allows comparisons between instrumented bike technologies. The study indicated that with few exceptions, riders of e-bikes behaved much the same as riders of regular bicycles. Violation rates were very high for both kinds of vehicles. Riders of regular bicycles and e-bikes both rode the wrong way on 45% and 44% of segments, respectively. Lu et al. [14] compared the risky behaviors of e-bike, e-scooter, and bicycle riders as they were crossing signalized intersections. They developed binary logit models to evaluate how variables affected the behaviors of two-wheeled vehicle riders at signalized intersections. Three different types of risky behaviors were identified, including stopping beyond the stop line, riding in motorized lanes, and riding against traffic. Chen et al. [15] summarized the four main types of moped conflicts divided by the conflict movement direction and their characteristics. They found that there were no significant differences in conflict severity between conflicts caused by violation behaviors and normal conflicts caused by phase shared.

Huertas-Leyva et al. [16] collected naturalistic data from six regular bicycle riders who each rode e-bikes during a period of two weeks, for a total of 32.5 h of data, and investigated how cyclists on e-bikes used front and rear brakes during routine cycling. They found out that in the majority of braking events during routine cycling, cyclists used only one brake at a time, favoring one of the two brakes according to a personal pre-established pattern. Yu et al. [17] studied electric bicycle riders’ responses to pedestrian countdown signal devices (PCSDs)—in particular, red light violations (RLVs) and early start behaviors. They used binary logit models to evaluate the influence of the associated factors. They found that PCSDs were effective in reducing the number of red-light running violations, and significant variables for RLV behaviors included being female, no pillion passengers, the type of electric bicycle, and the number of turning vehicles. Gao et al. [18] carried out a video-based observational study in Changsha, China to estimate the incidence of five unsafe bicycling behaviors among both shared and personal bike riders. They found that the incidences of not wearing a helmet, violating traffic lights, riding in the opposite direction of traffic, not holding the handlebar with both hands, and riding in a non-bicycle lane were 99.28%, 19.57%, 13.73%, 2.57%, and 64.06%, respectively.

Nurten et al. [19] investigated how design factors influence cyclist casualty severity at give-way roundabouts with mixed traffic. They found that a higher speed limit reduced the safety for cyclists at roundabouts and the probability of a serious casualty increased by about five times for each additional number of lanes on approach. Pulvirenti et al. [20] analyzed the behavior and safety of bicyclists on roundabouts with different diameters. The behavioral analysis revealed that, regardless of the type of condition, bicyclists were always faster on roundabouts with a large diameter and slower on roundabouts with a smaller diameter. Moreover, bicyclists were closer to the central island on roundabouts with a large diameter compared to roundabouts with a small diameter. Tim et al. [21] studied interactions between bicyclists and buses on shared bus lanes. They found that close interactions between bicyclists and buses were relatively frequent on both types of bus lanes and close overtaking and close bicycle-following were quite common. The overtaking speed of the buses was significantly higher on the wider bus lane compared to the narrower one. Farah et al. [22] analyzed drivers overtaking cyclists on rural roads. A driving simulator study was designed to assess driver decision-making during the overtaking. They found that the lateral comfort distance was mostly affected by the longitudinal distance between the subject vehicle and the oncoming vehicle, the longitudinal distance between the subject vehicle and the cyclist, and the presence of an oncoming vehicle.

From the literature survey discussed above, it can be observed that many researches have been conducted to explore the psychology, behaviors and movements of riders of non-motorized vehicles, using various methods. These studies are meaningful for the understanding of various behaviors, which could lead to efficient improvement of traffic safety. This paper aims to explore factors impacting on the lane choice of riders of non-motorized vehicles at exit legs of signalized at-grade intersections, which are critical to the efficient and safe performance of urban road systems.

## 3. Data Collection and Method

### 3.1. Data Collection

The lane choice of riders of non-motorized vehicles at exit legs of signalized at-grade intersections is affected by numerous factors. Some of the factors are subjective, such as emotion, habit, and disposition, which are very difficult to observe and estimate. For convenience of the research, only data concerning objective factors, which can be observed easily, were collected and analyzed by the research team.

After careful analysis of various elements concerning characteristics of riders, vehicles, roads, and traffic control, eight factors were considered to have some impact and were selected for the research. These factors are as follows: the sex and age of riders of non-motorized vehicles, type of non-motorized vehicles, movement of non-motorized vehicles, rate of time interval for non-motorized vehicles to enter the exit leg, volume of non-motorized vehicles at the exit leg, volume of motorized vehicles at the exit leg, and width of lane for non-motorized vehicles at the exit leg.

The sex of riders of non-motorized vehicles was recognized through careful observation of the appearance and clothing of each individual rider. Ages of riders were estimated by observers. To simplify the task of age estimation, riders of non-motorized vehicles were classified into three categories: young riders (12 to 24 years old), middle-aged riders (25 to 60 years old) and old riders (more than 60 years old). Field surveys indicated that non-motorized vehicles could be classified into the following three types: electric bicycles having two wheels and powered by electricity, electric tricycles having three wheels and powered by electricity and traditional bicycles having two wheels and powered by man. At an intersection, riders of non-motorized vehicles made one of three types of movements to enter the exit leg; the movements were as follows: right turns, left turns and through movements, which are easy to recognize.

The rate of time interval for non-motorized vehicles to enter the exit leg of a signalized at-grade intersection was calculated by dividing the time interval, during which it was permissible for non-motorized vehicles to enter the exit leg, by cycle length of the traffic signal. This factor was closely related with signal phasing and signal timing. The time interval, during which non-motorized vehicles could enter, were assigned to a specific phase which permitted vehicles to make some kinds of movements at one or more approaches of an intersection.

As there was only one lane for non-motorized vehicles at any exit leg, the volume of non-motorized vehicles at the exit leg was calculated by dividing the number of non-motorized vehicles entering the exit leg by the time interval during which the non-motorized vehicles were observed. The volume of motorized vehicles at the exit leg was calculated by dividing the number of motorized vehicles entering the exit leg by the time interval during which the motorized vehicles were observed and the number of lanes of the exit leg for motorized vehicles. The width of lane for non-motorized vehicles at the exit leg was measured by a flexible rule.

To select suitable sites that best satisfy the research objective and to control for the impacts of various confounding factors that affect the lane choice of riders of non-motorized vehicles, the following criteria were applied in the site selection process:

(1) The selected signalized at-grade intersections should be in or near residential areas. As non-motorized vehicles are mainly used for short-distance travel to satisfy the daily needs of inhabitants for commuting, shopping, entertainment and other purposes, it is more possible to observe large amounts of riders of non-motorized vehicles in or near such residential areas.
(2) The selected signalized at-grade intersections should be in a territory of flat terrain. Uneven ground usually takes riders of non-motorized vehicles greater effort, bringing about negative feelings and influencing their behaviors while crossing intersections. The influence of various types of terrain upon the lane choice of riders of non-motorized vehicles is rather complicated and has been excluded from the research.
(3) The selected signalized at-grade intersections should have no access connections in their functional areas. The functional area extends both upstream and downstream from the physical intersection area and includes the longitudinal limits of auxiliary lanes. This is the area where road users respond to the intersection, decelerating and maneuvering into the appropriate lane to stop or complete a turn. Access connections in the functional area can hamper the movements of vehicles and influence the lane choice of riders of non-motorized vehicles. The influence of access connections upon the lane choice of riders of non-motorized vehicles is rather complicated and is excluded from the research.
(4) At the exit legs of the selected signalized at-grade intersections, traffic volumes of non-motorized vehicles and motorized vehicles should have a wide range. Traffic volumes may be low during non-rush hours, while at rush hours, the intersections should experience high volumes. A wide range of traffic volumes makes it more suitable to analyze the impacts of traffic volumes upon the lane choice of riders of non-motorized vehicles.
(5) The selected signalized at-grade intersections should be formed by the intersecting of arterial roads (or streets) and/or collector roads (or streets). At such intersections, interactions between motorized vehicles and non-motorized vehicles are more obvious; therefore, they are more suitable for the analysis of impacts of motorized vehicles upon the lane choice of riders of non-motorized vehicles. On the other hand, local streets are obviously different from arterial and collector roads. They mainly serve pedestrians and non-motorized vehicles, and the movements and speeds of motorized vehicles are strictly controlled and limited. Therefore, at sites where local streets are involved, the impacts of motorized vehicles may be very small and not suitable for analysis.
(6) The selected signalized at-grade intersections should be cross-shaped. Most signalized at-grade intersections are cross-shaped or T-shaped. Compared with T-shaped intersections, at cross-shaped intersections, riders of non-motorized vehicles usually have all of the alternatives of movement: turning right, turning left, and going forward. However, at T-shaped intersections, riders of non-motorized vehicles have only two alternatives of movement, with going forward or turning left or right becoming impossible. Therefore, in order to study the impacts of movements of non-motorized vehicles upon the lane choice of their riders, it is better to select cross-shaped intersections.

Widespread field surveys were carried out in Nanjing, a populous and economically developed city in the east of China. After comprehensive analysis, four typical signalized at-grade intersections were finally selected for the research. All of the selected intersections are cross-shaped and are formed by intersection of a south-north road and an east-west road. Characteristics of exits legs of the four selected intersections are listed in Table 1.

For convenience of data collection, digital video cameras were used to record all the relevant vehicles at exit legs of the four signalized intersections. Later on, the research team played digital video files over and over again to obtain most of the data for the research, such as the lane choice, sex, and age of riders of non-motorized vehicles, type of non-motorized vehicles, movement of non-motorized vehicles, volume of non-motorized vehicles at the exit leg and volume of motorized vehicles at the exit leg.

It was noted that if the lane for non-motorized vehicles was very wide (5 m or more), riders of non-motorized vehicles were seldom observed to select the lane for motorize vehicles. Therefore, the research was confined to situations where the width of lane for non-motorized vehicles was less than 5 m. In total, 521 riders of non-motorized vehicles were observed and data concerning their choice of lanes and other impacting factors were collected and preliminarily analyzed. After dropping some data with drawbacks, 376 data were finally used for the analysis and model development.

### 3.2. Method

Statistical methods of regression analysis have been widely applied in the field of traffic engineering [23,24]. By analysis of quantitative relations between impacting factors and explained variables, regression analysis methods can be used to develop suitable mathematical models, which are very effective tools for the exploration of complicated traffic phenomena and help to identify underlying mechanisms. This paper was mainly concerned with the lane choice of riders of non-motorized vehicles, which had only two outcomes: the choice of lane for motorize vehicles and choice of lane for non-motorized vehicles.

For cases where there are two discrete outcomes, binary logistic regression can be applied to analyze the relationship between the dependent variable and independent variables. The dependent variable stands for the population proportion or probability (*P*) that the resulting outcome is equal to 1. The general form of the binary logistic regression can be expressed as:

(1) $$\log\left(\frac{P\left(Y=1|X_{1},X_{2},\cdots ,X_{p}\right)}{1-P\left(Y=1|X_{1},X_{2},\cdots ,X_{p}\right)}\right)=\alpha +\beta_{1}X_{1}+\beta_{2}X_{2}+\cdots +\beta_{p}X_{p},$$

where *Y* indicates the dichotomous dependent variable and has only two values: 1 and 0; $X_{i}$ indicate independent variables (i=1,2,⋯,p); $\beta_{i}$ are logistic regression coefficients  (i=1,2,⋯,p); and α is the intercept term.

A transformation of Equation (1) can be used to calculate the probability ratio and is given as:

(2) $$\frac{P\left(Y=1|X_{1},X_{2},\cdots ,X_{p}\right)}{1-P\left(Y=1|X_{1},X_{2},\cdots ,X_{p}\right)}=\exp\left(\alpha +\beta_{1}X_{1}+\beta_{2}X_{2}+\cdots +\beta_{p}X_{p}\right)\;,\;\;\;$$

Equation (2) indicates that when an independent variable, $X_{i}(i=1,2,\cdots ,p)$, increases by one unit, with all other independent variables remaining unchanged, the probability ratio increases by a factor $\exp(\beta_{i})$, which is called the odds ratio (the ratio of probability of occurrence of an event to the probability of non-occurrence of an event). The odds ratio ranges from zero to positive infinity and indicates the relative amount by which the odds of the outcome increase or decreases with the increase of an independent variable. More specifically, if coefficients are positive, for each additional unit increase in the variable $X_{i}$, the odds of *Y* = 1 is increased by $100\cdot \left[\exp\left(\beta_{i}\right)-1\right]$ percent. On the other hand, if coefficients are negative, a one-unit increase decreases the odds of *Y* = 1 by $100\cdot \left[1-\exp\left(\beta_{i}\right)\right]$ percent.

## 4. Results and Discussions

In the research, a probability prediction model for the lane choice of riders of non-motorized vehicles was developed to explore various factors impacting riders’ illegal usage of lanes allocated exclusively to motorized vehicles at exit legs of signalized at-grade intersections. The dependent variable of the model is the lane choice of riders of non-motorized vehicles with 1 for the choice of lane assigned to non-motorized vehicles and 0 for the choice of lane assigned to motorized vehicles. As has been discussed above, eleven independent variables for the eight impacting factors were initially taken into consideration. The descriptive statistics of these variables are summarized in Table 2. Among 376 riders of non-motorized vehicles, 141 riders illegally chose lanes for motorized vehicles, which accounted for 37.5%. This indicated that the problem of the illegal usage of lanes assigned to motorized vehicles is quite serious. This phenomenon of widespread illegal usage of lanes for motorized vehicles is supported by Gao et al. [18], who found that riding on a motorway accounted for 44.06% of all recorded cyclists in situations where a bicycle lane was available.

In order to choose the significant variables from the 11 independent variables listed in Table 2, a forward stepwise variable selection method with likelihood-ratio removal criterion was used. Four variables were found to be statistically insignificant and were excluded from the model. The best model has seven independent variables, of which three are continuous variables and four are dummy variables, and the results are given in Table 3. The final equation for probability prediction of non-motorized vehicle rider’s choice of lane assigned to non-motorize vehicles is given as follows:

(3) $$P\left(Y=1\right)=\frac{\exp\left(-0.985-0.736\;X_{1}+1.243X_{2}+2.730X_{3}-2.497X_{4}-0.00722X_{5}+0.00175X_{6}+0.665X_{7}\right)}{1+\exp\left(-0.985-0.736\;X_{1}+1.243X_{2}+2.730X_{3}-2.497X_{4}-0.00722X_{5}+0.00175X_{6}+0.665X_{7}\right)},\;\;\;$$

where *Y* is the choice of riders of non-motorized vehicles (1 for the choice of lane assigned to non-motorized vehicles, 0 for the choice of lane assigned to motorized vehicles), and the independent variables are explained in Table 3.

Table 3 summarizes the output of binary logistic regression analysis. All of the independent variables selected for the model are statistically significant. Coefficients for these variables can be used to analyze the impacts of various factors upon probability of lane choice of riders of non-motorized vehicles.

The coefficient for the indicator variable for the sex of rider of a non-motorized vehicle is negative, indicating that male riders of non-motorized vehicles are less likely to choose the lane for non-motorized vehicles at the exit leg of a signalized at-grade intersection. The exponential of the coefficient B of this variable (0.479) expresses the odds ratio (the ratio of probability of occurrence of an event to the probability of non-occurrence of an event). Based on the value, if other factors remain the same, compared with female riders, male riders will result in a 1 − 0.479 = 52.1% decrease in the odds that the lane assigned to non-motorized vehicles is chosen by riders of non-motorized vehicles, assuming that all other factors remain constant. This implies that, as far as the lane choice of riders of non-motorized vehicles is concerned, the behaviors of male riders of non-motorized vehicles are riskier than those of female riders because they tend to choose the lane for motorized vehicles instead of the lane for non-motorized vehicles more frequently, though such behaviors are illegal and dangerous. This finding is in agreement with Wang et al. [10], who studied the risky driving behavior of drivers of electric bicycles by the analysis of questionnaires and found that male drivers had more frequent illegal behaviors.

The coefficient for the indicator variable for the type of non-motorized vehicle is positive, implying that riders of different types of non-motorized vehicles tend to behave differently, as far as the choice of lane is concerned. Riders of traditional bicycles are more likely to choose the lane for non-motorized vehicles than riders of electric bicycles and tricycles. This means that riders of non-motorized vehicles powered by electricity are more likely to choose the lane for motorized vehicles. Based upon the value of the odds ratio (3.466), compared with traditional bicycles, electric bicycles and electric tricycles will result in a 3.466 − 1 = 246.6% increase in the odds that the lane for motorized vehicles is chosen by riders of non-motorized vehicles, assuming that all other factors remain constant. This big difference may be explained by both the better performance of electric bicycles and tricycles and the relative narrowness of the lane for non-motorized vehicles. With electricity to provide power, riders of non-motorized vehicles can accelerate to rather higher speeds easily. Their intentions to accelerate, however, are often hampered by other slow-moving vehicles, and the narrowness of the lane for non-motorized vehicles can make the situation even worse. In order to achieve higher speeds at great ease, some imprudent riders of electric bicycles and tricycles may be induced to use the adjacent lane for motorized vehicles, which are usually wider and offer more chances for higher speeds, in spite of the fact that they may encounter great danger when they are involved in conflicts with motorized vehicles. The finding concerning the risky behavior of riders of electric bicycles was supported by Chen et al. [9], who found that the power-assisted bicycle riders reported significantly higher ratios of traffic safety-related risk behaviors.

**Figure 2:**
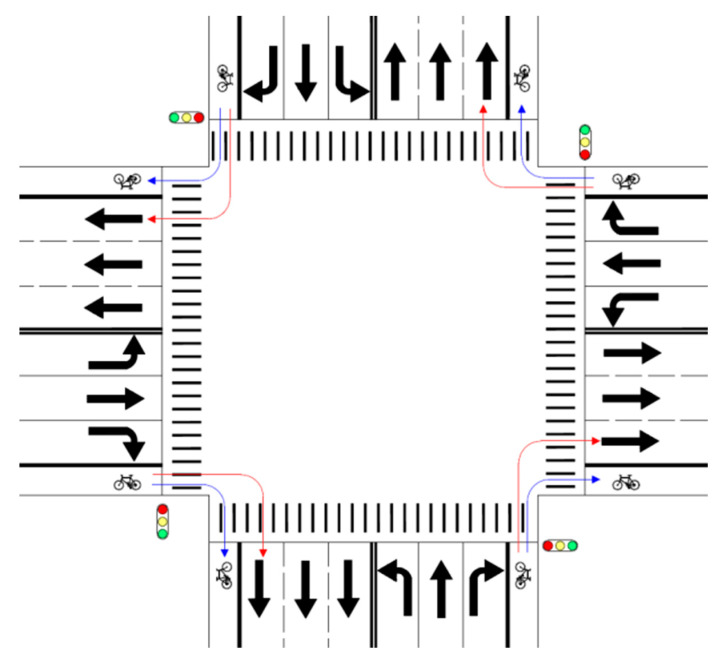
Trajectories of right-turning non-motorized vehicles with blue lines for choice of lane for non-motorized vehicles and red lines for choice of lane for motorized vehicles.

The coefficient for the indicator variable for the right turn movement of non-motorized vehicle is positive. This indicates that when performing a right turn movement to enter the exit leg of a signalized at-grade intersection, a rider of a non-motorized vehicle is more likely to choose the lane for non-motorized vehicles. Based upon the value of the odds ratio (15.338), compared with through movement, right turn movement will result in a 15.338 − 1 = 1433.8% increase in the odds that the lane for non-motorized vehicles is chosen by riders of non-motorized vehicles, if all other factors remain unchanged. This huge difference may be explained by the obvious reason that it will be rather more difficult for riders of non-motorized vehicles to perform right turn movements if they choose the lane for motorized vehicles (see Figure 2). As the lane for motorized vehicles is to the left of the lane for non-motorized vehicles at the exit leg, right-turning riders of non-motorized vehicles have to travel a longer distance and encounter more conflicts with other vehicles if the lane for motorized vehicles is chosen. Besides, such behaviors may be hampered by riders making through or left-turning movements, who have to wait for green lights at stop lines before entering the intersection. The situations discussed above will usually need right-turning riders to pay more attention and takes more effort, and therefore discourages them from choosing the lane for motorized vehicles.

**Figure 3:**
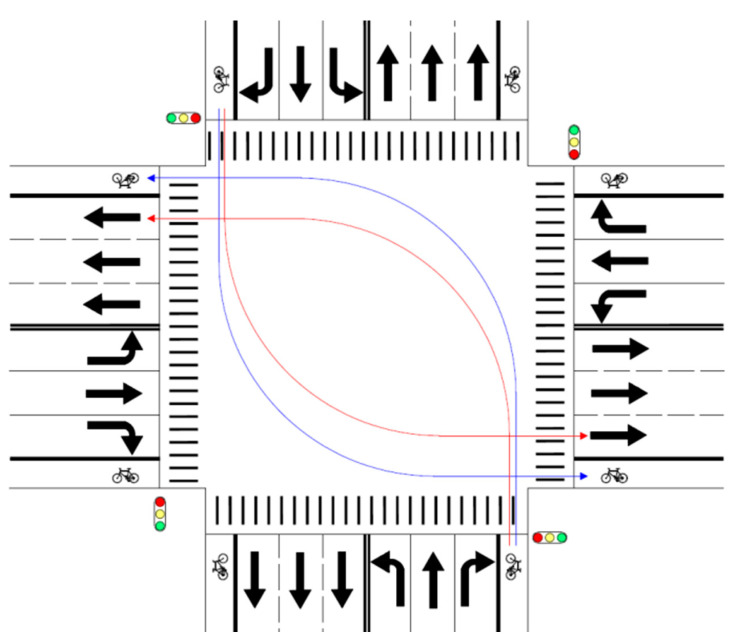
Trajectories of left-turning non-motorized vehicles with blue lines for choice of lane for non-motorized vehicles and red lines for choice of lane for motorized vehicles.

The coefficient for the indicator variable for the left turn movement of a non-motorized vehicle is negative, indicating that when performing a left turn movement to enter the exit leg of a signalized at-grade intersection, a rider of a non-motorized vehicle is less likely to choose the lane for non-motorized vehicles. Based upon the value of the odds ratio (0.823), compared with through movement, left turn movement will result in a 1 − 0.823 = 17.7% decrease in the odds that riders of non-motorized vehicles will choose the lane for non-motorized vehicles, assuming that all other factors remain the same. This phenomenon may be explained by the reason that it will be easier for riders of non-motorized vehicles to perform left turn movements if they choose the lane for motorized vehicles (see Figure 3). As the lane for motorized vehicles is to the left of the lane for non-motorized vehicles at the exit leg, left-turning riders of non-motorized vehicles need to travel a shorter distance and encounter fewer traffic conflicts if the lane for motorized vehicles is chosen. Such situations may induce riders of non-motorized vehicles with a risk preference to choose the lane assigned to motorized vehicles, though their choices are illegal and dangerous.

The coefficient for the volume of non-motorized vehicles at the exit leg is negative, which indicates that the probability of choosing the lane for non-motorized vehicles decreases with an increase of volume of non-motorized vehicles at the exit leg. More specifically, when the volume of non-motorized vehicles at the exit leg increases, riders of non-motorized vehicles are less likely to choose the lane for non-motorized vehicles. The value of the odds ratio (0.9928) means that a one-unit increase in volume of non-motorized vehicles at the exit leg leads to a 0.72% decrease in the odds of choosing the lane for non-motorized vehicles. As the volume of non-motorized vehicles at the exit leg increases, riders of non-motorized vehicles will find that they are surrounded more closely by other non-motorized vehicles, which usually causes uncomfortable feelings because of shrinkage of private space. With a higher volume of non-motorized vehicles, it also becomes more difficult for riders of non-motorized vehicles to reach a faster speed. Under such situations, riders with a risk preference may be induced to choose the lane for motorized vehicles instead of the lane for non-motorized vehicles.

The coefficient for the volume of motorized vehicles at the exit leg is positive, implying that the probability of choosing the lane for non-motorized vehicles will increase when the volume of motorized vehicles at the exit leg increases. That is to say, as the volume of motorized vehicles at the exit leg increases, more riders of non-motorized vehicles are observed to choose the lane for non-motorized vehicles. The value of the odds ratio (1.0018) means that a one-unit increase in the volume of motorized vehicles at the exit leg leads to a 0.18% increase in the odds choosing the lane for non-motorized vehicles. When the volume of motorized vehicles at the exit leg increases, distances between motorized vehicles will become smaller, and it will be harder to find a large enough gap in traffic stream of motorized vehicles for riders of non-motorized vehicles, who plan to choose the lane for motorized vehicles. As a result, more riders of non-motorized vehicles will have to choose lane for non-motorized vehicles.

The coefficient for the width of the lane for non-motorized vehicles is positive, indicating that the probability of choosing the lane for non-motorized vehicles increases with an increase of width of the lane for non-motorized vehicles. This means that when the width of the lane for non-motorized vehicles increases, riders of non-motorized vehicles are more likely to choose the lane for non-motorized vehicles. The value of the odds ratio (1.945) indicates that a one-unit increase in the width of the lane for non-motorized vehicles leads to a 94.5% increase in the odds of choosing the lane for non-motorized vehicles. This phenomenon implies that riders of non-motorized vehicles are very sensitive to the width of lane for non-motorized vehicles at exit legs of signalized at-grade intersections. With a wider lane for non-motorized vehicles, riders have more space and feel more comfortable. Many kinds of maneuvers, such as accelerating, decelerating, overtaking, left-turning, and right-turning, are much easier. Under such circumstances, riders of non-motorized vehicles will become more willing to use the lane for non-motorized vehicles.

Later on, the performance of the binary logistic model was analyzed, based upon the data for model calibration. The prediction model was used to predict a rider’s choice of lane, given the values of independent variables. Correctness of prediction for the lane choice of each of the 376 riders was checked. The results are summarized in Table 4, which indicates that the model is acceptable. For the choice of lane assigned to motorized vehicles (Y = 0), the percentage of correct predictions is 54.61%. For choice of lane assigned to non-motorized vehicles (Y = 1), the percentage of correct predictions is 89.79%. The overall percentage of correct predictions is 76.60%. The analysis discussed above indicates that predictions of the model are in good agreement with the observed facts. Therefore, the model can be used to predict how various factors impact on the lane choice of riders of non-motorized vehicles at exit legs of signalized at-grade intersections.

Probability curves of the lane choice of riders of non-motorized vehicles can be drawn to show how various factors influence the lane choice of riders at exit legs of signalized at-grade intersections by using Equation (3). Before drawing the curves, a series of calculations of probability of choosing the lane for non-motorized vehicles should be carried out by giving one independent variable a series of different values while keeping all the other independent variable constant. For this purpose, assuming the volume of motorized vehicles to be at the average level (465 vehicles/h), the probability of choosing the lane for non-motorized vehicles is depicted as a function of volume of non-motorized vehicles at the exit leg (holding all the other independent variables at a fixed value).

**Figure 4:**
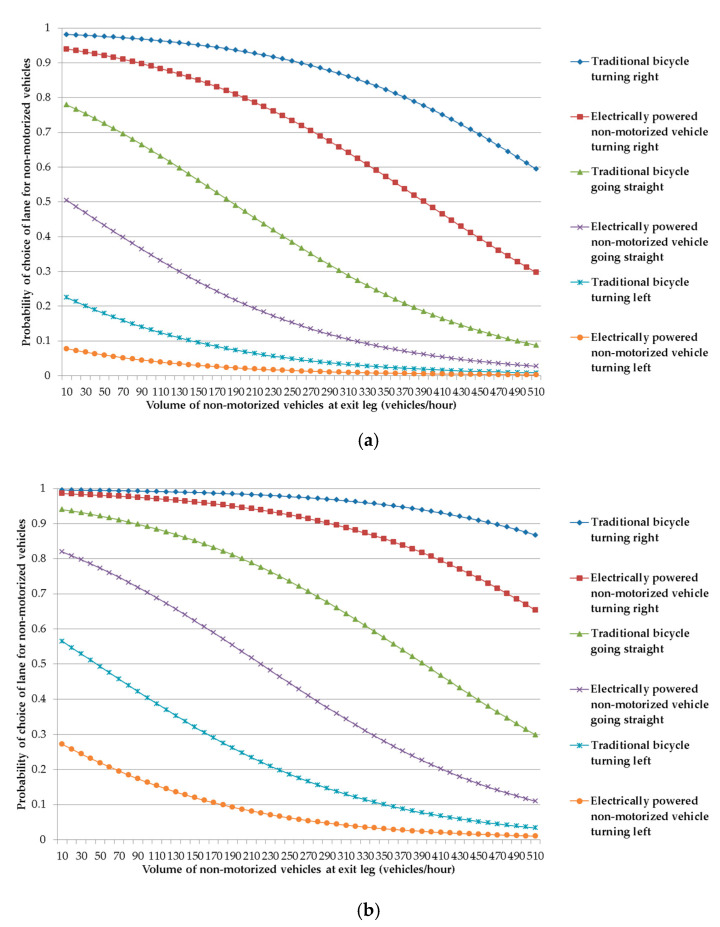
Probability curves of lane choice of male riders of non-motorized vehicles. (**a**): Width of lane for non-motorized vehicles being 1.5 m; (**b**): width of lane for non-motorized vehicles being 3.75 m.

**Figure 5:**
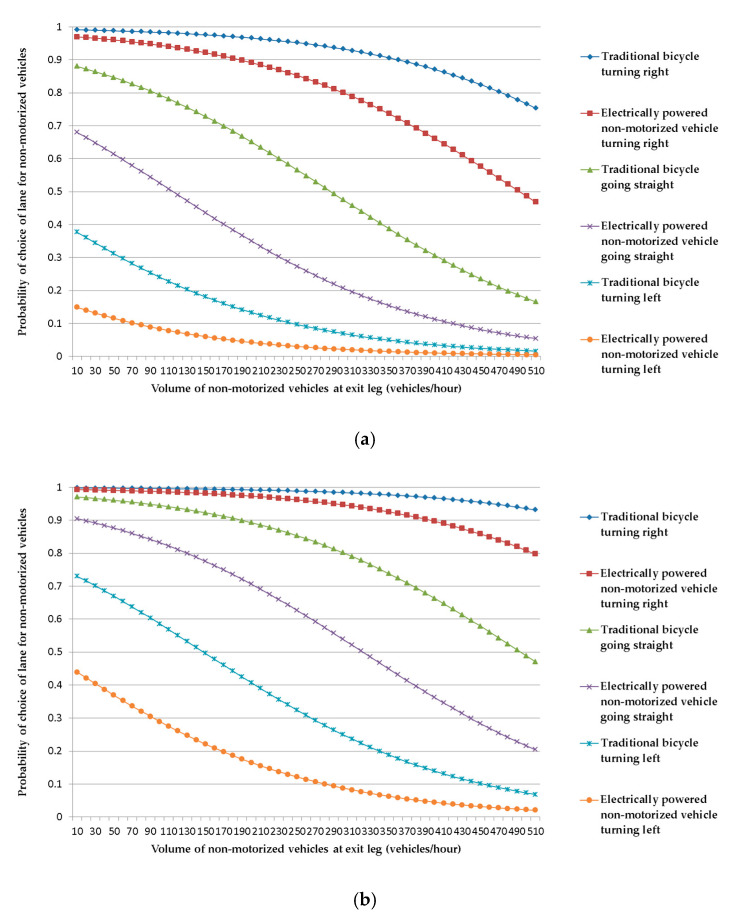
Probability curves of lane choice of female riders of non-motorized vehicles. (**a**): Width of lane for non-motorized vehicles being 1.5 m; (**b**): width of lane for non-motorized vehicles being 3.75 m.

The research team was especially concerned about the influences of the characteristics of non-motorized vehicles and their riders. Dummy variables concerning such characteristics as the type of non-motorized vehicle, movement of non-motorized vehicles and sex of the riders of non-motorized vehicles were given different values to represent various specific circumstances. A series of curves were drawn to show these influences, as shown in Figure 4 and Figure 5. All of the probability curves show a common trend, which indicates that the probability choosing the lane for non-motorized vehicles decreases with an increase in the volume of non-motorized vehicles at the exit leg, assuming that all of the other explanatory variables (impacting factors) remain constant. This conclusion is in agreement with what has been discussed above about the coefficient for the volume of non-motorized vehicles at the exit leg.

Figure 4 can be used to explore the characteristics of the lane choice of male riders of non-motorized vehicles. The probability curve for traditional bicycles turning right is above all of the other probability curves, and it is closely followed by the probability curve for electrically powered non-motorized vehicles turning right. The next two probability curves are those for traditional bicycles going straight and electrically powered non-motorized vehicles going straight. The last two probability curves are those for traditional bicycles turning left and electrically powered non-motorized vehicles turning left. It is easy to see that for male riders of non-motorized vehicles, the impact of movement of non-motorized vehicles is rather bigger than that of the type of non-motorized vehicle. No matter what type of non-motorized vehicle may be concerned, the probability curve for turning right is above the probability curve for going straight, which, in turn, is above the probability curve for turning left. For the same movement of non-motorized vehicles, the probability curve for traditional bicycles is above that for electrically powered non-motorized vehicles.

It is obvious that the probability curves of Figure 4a and probability curves of Figure 4b have similar characteristics. However, a close comparison of the probability curves of Figure 4b with those of Figure 4a indicates that for the same volume of non-motorized vehicles at the exit leg, the probability of choosing the lane for non-motorized vehicles on any curve of Figure 4b is bigger than that on the corresponding curve of Figure 4a, implying that a wider lane for non-motorized vehicles induces more riders to choose the lane for non-motorized vehicles, no matter which types of non-motorized vehicles are used and which movements are made. Furthermore, a careful inspection of the curves of Figure 4a and the corresponding curves of Figure 4b indicates that the impact of the lane width for non-motorized vehicles upon the probability of choosing the lane for non-motorized vehicles is much bigger for such movements as going straight and turning left when the volume of non-motorized vehicles at the exit leg is relatively low.

The probability curves of Figure 5 show how various factors influence the lane choice of female rider of non-motorized vehicle. Comparison of the probability curves of Figure 5 with those of Figure 4 indicates that factors such as the type of non-motorized vehicle, movement of non-motorized vehicle, and volume of non-motorized vehicles at the exit leg impact on the choice of lane of female riders in similar ways as they impact on that of male riders. The main difference is that when all of the other factors are the same, female riders have a greater probability of choosing the lane for non-motorized vehicles than male riders.

## 5. Conclusions

Due to their vulnerability, risky behaviors of riders of non-motorized vehicles are one of the major causes of serious traffic accidents. The control or reduction of such risky behaviors are therefore of great importance. Based upon data collected from four typical signalized at-grade intersections, this research has explored riders’ lane choice at exit legs of signalized intersections, and a probability prediction model has been developed, including seven independent variables, which are concerned with many different aspects, such as the characteristics of riders and non-motorized vehicles, geometric design of roads, and traffic volumes. The results of this research can assist researchers and practitioners in understanding the mechanisms underlying illegal behaviors of riders of non-motorized vehicles about their choice of lane at exit legs of signalized at-grade intersections. The probability prediction model can be used to help riders to behave more safely.

The model can be used to quantify the influences of various explanatory variables on the probability of choosing the lane for non-motorized vehicles, and the impacts can be shown graphically by using probability curves, which are based upon calculations of the prediction model. Designers can use the model to evaluate how geometric designs, such as the number of lanes for motorized vehicles at the exit leg and the width of lane for non-motorized vehicles at the exit leg, influence riders of non-motorized vehicles at the exit leg of signalized at-grade intersections. It is hopeful that the research will help transportation decision-makers to develop technical guidelines governing the design and management of signalized at-grade intersections.

Improvements need to be carried out in order to rectify the limitations in the research when further studies are to be carried out. First of all, it should be pointed out that because the data of the research were relatively limited, similar studies should be carried out in other places to check the results. Explanations of the reasons for the impacting factors were not based upon subjective reports of riders of non-motorized vehicles, and therefore their correctness needs to be testified in the future. Thirdly, the four variables, which were not included in the prediction model, may have some influence on riders’ behaviors. Because their statistical insignificance may be due to the limitation of the data, researchers are encouraged to analyze the factors represented by these excluded variables in the future. Fourthly, as well as the volume of motorized vehicles, the speed of motorized vehicles can also be taken into consideration when the choice of lane of riders of non-motorized vehicles are explored because fast-moving motorized vehicles can have a great impact on the mental activities of riders of non-motorized vehicles, which might have some influence upon riders’ behavior. At last, other behaviors of riders of non-motorized vehicles at exit legs of intersections, such as lane-changing and overtaking, should be explored in order to have a more complete understanding of their complicated behaviors.

## Figures and Tables

**Table 1:** Characteristics of exit legs of the four selected intersections.

Intersection Number		1	2	3	4
Eastbound Exit leg	Number of Lanes for Motorized Vehicles	1	1	1	3
	Width of Lane for Non-Motorized Vehicles (m)	1.50	3.75	3.75	5.00
Westbound Exit leg	Number of Lanes for Motorized Vehicles	1	1	1	3
	Width of Lane for Non-Motorized Vehicles (m)	3.75	3.75	3.75	5.00
Southbound Exit leg	Number of Lanes for Motorized Vehicles	4	1	1	1
	Width of Lane for Non-Motorized Vehicles (m)	3.50	3.00	3.50	3.50
Northbound Exit leg	Number of Lanes for Motorized Vehicles	4	1	1	1
	Width of Lane for Non-Motorized Vehicles (m)	3.50	3.00	3.50	3.50

**Table 2:** Descriptive statistics of variables for model development.

Variables	Min	Max	Mean	Standard Deviation	Frequency
Lane Choice of Non-Motorized Vehicle Riders	—	—	—	—	376
1 (Choice of Lane for Non-Motorized Vehicles)	—	—	—	—	235 (62.5%)
0 (Choice of Lane for Motorized Vehicles)	—	—	—	—	141 (37.5%)
Sex of Riders	—	—	—	—	376
1 (Male)	—	—	—	—	268 (71.3%)
0 (Female)	—	—	—	—	108 (28.7%)
Age of Riders	—	—	—	—	376
0 (Young)	—	—	—	—	154 (41.0%)
1 (Middle-aged)	—	—	—	—	208 (55.3%)
2 (Old)	—	—	—	—	14 (3.7%)
Type of Non-Motorized Vehicles	—	—	—	—	376
0 (Electric Bicycles)	—	—	—	—	336 (89.4%)
1 (Electric Tricycles)	—	—	—	—	12 (3.2%)
2 (Traditional Bicycles)	—	—	—	—	28 (7.4%)
Movement of Non-Motorized Vehicles	—	—	—	—	376
0 (Through Movement)	—	—	—	—	238 (63.3%)
1 (Right Turn)	—	—	—	—	81 (21.5%)
2 (Left Turn)	—	—	—	—	57 (15.2%)
Rate of Time Interval for Non-Motorized Vehicles to Enter Exit Leg	0.12	1.00	0.56	0.24	376
Volume of Non-Motorized Vehicles at Exit Leg (vehicles/h)	35	220	127.33	66.9	376
Volume of Motorized Vehicles at Exit Leg (vehicles/h)	113	758	464.83	162.15	376
Width of Lane for Non-Motorized Vehicles (m)	1.5	3.75	3.19	0.74	376

—: not applicable.

**Table 3:** Binary logistic model for lane choice of riders of non-motorized vehicles.

Variables		Significance	OR	95% Confidence Interval for OR	
				Lower Bound	Upper Bound
X_1_	Sex of Rider of Non-Motorized Vehicle (Male)	0.02	0.48	0.26	0.87
X_2_	Type of Non-Motorized Vehicle (Traditional Bicycle)	0.03	3.47	1.17	10.27
X_3_	Right Turn Movement of Non-Motorized Vehicle;	0.00	15.34	4.93	47.68
X_4_	Left Turn Movement of Non-Motorized Vehicle;	0.00	0.82	0.03	0.20
X_5_	Volume of Non-Motorized Vehicles at Exit Leg (vehicles/h)	0.01	0.99	0.99	1.00
X_6_	Volume of Motorized Vehicles at Exit Leg (vehicles/h)	0.07	1.00	1.00	1.00
X_7_	Width of Lane for Non-Motorized Vehicles (m)	0.02	1.95	1.10	3.43
	Constant	0.49	0.37	—	—

—: not applicable.

**Table 4:** Classification table for analysis of model performance.

Observed Choice of Lane	Estimated Choice of Lane		Percentage of Correct
	0	1	
0	77	64	54.61%
1	24	211	89.79%
Overall Percentage of Correct	---	---	76.60%

## Data Availability

The data presented in this study are available on request from the corresponding author. The data are not publicly available due to the requirement of our funder.
